# Unit one is intrusive

**DOI:** 10.1088/1681-7575/ad4bea

**Published:** 2024-06

**Authors:** David Flater

**Affiliations:** Information Technology Laboratory, National Institute of Standards and Technology, Gaithersburg, MD, United States of America

**Keywords:** unit one, dimensionless quantity, SI

## Abstract

The SI brochure’s treatment of quantities that it regards as dimensionless, with the associated unit one, requires certain physical quantities to be regarded as simply numbers. The resulting formal system erases the nature of these quantities and excludes them from important benefits that quantity calculus provides over numerical value calculations, namely, that accidental confusion of different units and different kinds of quantities is sometimes prevented. I propose a better treatment that entails removing from the SI brochure those prescriptions that conflict with common practices in the treatment of dimensionless quantities, especially the definition and use of non-SI dimensionless units that are distinguished by kind.

## 1. Introduction

A quantity in the International System of Units (SI) can be stated as the product of a numerical value and a unit of measurement. The unit is derived from the seven SI base quantities, which measure seven different physical dimensions. However, many kinds of quantities—all ratios of two quantities of the same kind, all counted quantities (numbers of entities or events), all ‘characteristic numbers’, and all products where the dimensions cancel out—have no extent in any of those dimensions and are thus called ‘dimensionless quantities’ (among other names).

The unit that the SI brochure [1] identifies for dimensionless quantities is the special unit one, which behaves as a multiplicative identity element. One might assume that unit one is an algebraic formality with no operational impact, but this is not the case. Unit one is *intrusive*; i.e. its presence as the unit associated with a quantity is unwelcome and inconvenient. By invisibly occupying the place where a meaningful unit could otherwise go, it prevents us from treating counted quantities as anything more than plain numbers and deprives us of advantages that quantity calculus has over purely numerical calculations (unless we disregard the prescriptions of the SI brochure).

The following sections provide the details supporting this claim, a review of related work in the literature, an assessment, and finally an outline of changes to the SI brochure that would mitigate the problems.

## 2. Unit one in SI

Editions 1 through 6 of the SI brochure did not call unit one by name but included a note on the subject of ‘dimensionless quantities’ or ‘quantities expressed as pure numbers’ that changed little from 1970 to 1991. The note did not specifically address counted quantities. The following is the English text from the 6th edition: ‘Certain so-called dimensionless quantities, as for example refractive index, relative permeability, or friction factor, are defined as the ratio of two comparable quantities. Such quantities have a dimensional product—or dimension—equal to 1 and are therefore expressed by pure numbers. The coherent SI unit is then the ratio of two identical SI units and may be expressed by the number 1.’ Notice that the final sentence does not name the unit one; it only mentions the number 1 as an expression, i.e. a unit symbol.

In the 7th edition (1998), the previous note expanded into a section (section 2.2.3) that explicitly named one as a coherent SI unit and listed three categories of quantities that are referred to it: ratios of two quantities of the same kind, ‘characteristic numbers’, and ‘numbers which represent a count’. ‘All of these quantities are described as being dimensionless, or of dimension one, and have the coherent SI unit 1. Their values are simply expressed as numbers and, in general, the unit 1 is not explicitly shown. In a few cases, however, a special name is given to this unit, mainly to avoid confusion between some compound derived units. This is the case for the radian, steradian, and neper.’ Unit one was referenced explicitly in tables 2 and 3 and in table footnotes.

The 8th edition (2006) discussed unit one in three separate sections, elaborating the narrative of the 7th edition.

- Section 1.3 addressed unit one in the context of dimensional products. For derived quantities where the dimensional exponents are all zero, ‘The coherent derived unit… is always the number one, 1’. Counted quantities ‘cannot be described in terms of the seven base quantities of the SI at all’ but ‘are also usually regarded as dimensionless quantities, or quantities of dimension one, with the unit one, 1.’
- Section 2.2.3 said that one is both a base unit and a derived unit. ‘Certain quantities are defined as the ratio of two quantities of the same kind. …There are also some quantities that are defined as a more complex product of simpler quantities in such a way that the product is dimensionless. …For all these cases the unit may be considered as the number one, which is a dimensionless derived unit.’ But counted quantities ‘are taken to have the SI unit one, although the unit of counting quantities cannot be described as a derived unit expressed in terms of the base units of the SI. For such quantities, the unit one may instead be regarded as a further base unit.’
- Section 5.3.7 added a prohibition on writing unit one explicitly except where a ‘special name’ has been accepted for it (i.e. units for angles and logarithmic ratio quantities): ‘The unit symbol 1 or unit name ‘one’ are not explicitly shown, nor are special symbols or names given to the unit one, apart from a few exceptions as follows.’ It also discussed the meaning of percent, parts per million, and the like.

The 9th edition (2019) retained essentially the same role for unit one but significantly changed its definition. Text addressing whether unit one is base or derived was removed as was text explicitly naming one as an *SI* unit. Instead, section 2.3.3 said ‘The unit one is the neutral element of any system of units—necessary and present automatically. There is no requirement to introduce it formally by decision. Therefore, a formal traceability to the SI can be established through appropriate, validated measurement procedures.’

Section 2.3.3 avoided directly describing angles or counted quantities as ‘just numbers’. Nevertheless, counted quantities were said to be ‘just numbers’ in section 5.4.7. Section 5.4.7 also introduced the option to use unsimplified unit expressions such as m/m to express ratios of two quantities of the same kind.

The text of the 9th edition has been updated several times. This letter refers specifically to version 2.01, updated December 2022 [2].

## 3. Unit one in the literature

In 1822, Fourier introduced the concept of physical dimension, thereby creating the distinction between dimensional and dimensionless quantities [3, art. 160].

In 1933, Busemann introduced a classification system for physical quantities in which dimensionless quantities were grouped into Class 0 (‘0. Klasse’) [4]. Class 0 quantities were those that he regarded as being measurable without first establishing an arbitrary scale. For counted quantities, the unit of counting implicitly established the scale; for angles, the cycle filled the same role. He concluded that Class 0 quantities can be described as pure numbers.

In 1955, unit one appeared as ‘die Einheit ‘Eins” in a book by Stille [5, I.8]. After introducing the number one as an effective unit, Stille immediately discussed the practice of adding designations to various quantities that are nominally expressed in unit one to distinguish them from one another. Stille regarded these designations as arithmetically equivalent to one, foreshadowing the SI brochure’s description of them as ‘special names’ for the unit one. He included a version of the widely-quoted equation where a dimensional product is apparently equated to the number 1 or to a dimension that the number 1 is meant to represent: $\text{Dim}\left[Y\right]=\prod_{i=1}^{n}{\text{A}}_{i}^{0}=1$. He noted also that the term Dimension Zero (‘Dimension Null’) was already in use.

In 1981, Reichardt rejected the position that numerical quantities (‘Zahlengrößen’), elsewhere called dimensionless quantities, are ‘just numbers’ [6]:

Numerical quantities are measurable properties of objects or processes, that is to say physical quantities, not ‘just numbers’. …Replacing a numerical quantity by a number is only permissible when numerically evaluating an equation connecting numerical quantities. It amounts to renouncing the qualitative properties of the particular quantity. It is not possible to deduce from mathematical derivations whether it is possible to assign qualitative properties to the resulting values. For this one has to resort to the physical considerations which gave rise to formulating and solving the equations in the first place. Therefore all symbols representing numerical quantities are containers for pure quantities, not for numbers. This is the only way in which the combinations which led to the formulation and sub-sequent treatment of the equations connecting the quantities adequately mirror the physical connections.

While recognizing that the names and symbols were a matter of convention and unimportant from a mathematical perspective, Reichardt used Dimension 1 as the working name for the dimension of numerical quantities and reported that East German standards had used 1 as the symbol for the corresponding unit.

In 1995, J de Boer wrote, ‘All the so-called ‘dimensionless quantities’ belong to one class of quantities of the same kind; they belong to the equivalence class of ‘dimensionless’ quantities’ [7, section A3.4]. ‘These dimensionless quantities are pure numbers’ [7, section 2.1.4].

Some pages later in the same journal issue, Mills reviewed ‘arguments for regarding the number 1 as a unit of the SI’ [8]. Mills addressed both counted quantities and ratios of two quantities of the same kind while de Boer addressed only the ratios.

Also in 1995, Giacomo criticized the term ‘dimension one’, pointing out that the analogy with *x*^0^ = 1 where *x* is a real number is unjustified for dimensions [9].

In 1998, Quinn and Mills recommended adoption of the special name uno (symbol U) for unit one to enable SI prefixes to be used instead of percent, parts per million, and suchlike, noting that it had been discussed in the Consultative Committee for Units (CCU) [10]. The article drew a comment from White and Nicholas saying that the problem ‘is fundamentally one of a lack of education, and not of a flaw in the SI’ [11].

In 2001, Mills, Taylor, and Thor looked at the definitions of the units radian, neper, bel, and decibel [12]. They made the following general comments on units for dimensionless quantities: ‘It is clear that as the value of a dimensionless quantity is always simply a number, the coherent unit… is always the number one, symbol 1. It is none the less important to establish the equation defining the quantity concerned… in order that the meaning of the coherent unit one can be correctly interpreted. …For all of them the coherent unit is one. However the significance of this unit is different for different quantities.’ The article drew a response from Emerson saying, ‘The concept of units for dimensionless quantities, whether they be the number one or other numbers, lacks convincing supporting logic’ and ‘Angle should instead be adopted formally as a base quantity with a definition that is consistent with the general, universal view of lexicographers and of the majority who understand and use the term in its classical sense’ [13].

In 2002, Valdés opposed expansion of the treatment of dimensionless quantities in SI: ‘Adopting special names for other dimensionless quantities, even another general special name for the number one, will add more confusion to that already existing’ [14].

In 2004, Dybkaer reviewed the previous history of unit one and the uno and asserted “Number of entities’ is dimensionally independent of the current base quantities and should take its rightful place among them’ [15]. He reported that the International Committee for Weights and Measures had decided not to act on the CCU’s recommendation of the uno.

Later in 2004, Emerson criticized the concept of unit one, concluding ‘If countable quantities, each with its own unit, are also recognized as base quantities, nothing remains of the case for *one* as a practical unit’ [16]. He rejected de Boer’s assertion (quoted above) in a footnote:

A unit of a quantity is necessarily a quantity of the same kind. Every kind of dimensionless quantity, *were it to have a unit*, would have to have a unit of its own kind, even if it bore the same name as the putative units of all other kinds of dimensionless quantities, notably *one*. Dimensionless quantities of all kinds are evaluated as numbers and are at the same time quantities of different kinds. The magnitude of a relative area, for example, cannot be meaningfully compared with that of a Reynolds number.

In 2010, Johansson proposed a parametric unit one—essentially, one of *something* that must be specified [17].

In 2013, Mitrokin introduced ‘[1]’ (the number 1 in square brackets, not bibliographic reference 1!) as a symbol for the ‘dimensional 1’ to distinguish it from the number 1 [18].

In 2015, for counted quantities, Brown and Brewer proposed explicitly writing 1 as a unit instead of omitting it, together with a full description of what is being counted [19].

Around the same time, Mohr and Phillips argued that radians and steradians should not be algebraically eliminated and advocated the introduction of counting units that allow clearer definition of both becquerel and amounts of substances [20]. The article drew a comment from Leonard alleging a number of ‘errors’ [21], in response to which Mohr and Phillips emphasized that they were proposing to change the definitions that Leonard was citing [22].

Also in 2015, Krystek distinguished the multiplicative neutral element in dimensional algebra from the number 1 (cf. Reichardt) and reiterated Giacomo’s criticism of the term ‘dimension one’: ‘the symbol ‘1’ denotes the number ‘one’ and ‘*x*^0^ = 1’ is only valid, if *x* actually represents a number. A dimensional product is however clearly *not* a number’ [23]. He proposed referring to dimension number with symbol Z and the coherent unit 1 instead of saying ‘dimension one’ or ‘dimensionless’ and described the values *of* the quantity number as pure numbers. ‘The quantity dimension Z is *not* a base dimension, like [the seven enumerated in SI], but rather the neutral element needed in any system of quantity dimensions.’

In 2016, Mills argued that the radian and the cycle should be treated as units of dimension angle, thereby clarifying that certain pairs of definitions refer to the same quantity expressed with different units [24]. Discussion of proposals to add dimension angle continued as a separate thread from the treatment of dimensionless quantities and has remained active [25–31].

In 2017, Flater proposed to use dimensionless units like those introduced by Mohr and Phillips to discern different kinds of dimensionless quantities in quantity calculus [32]. He arranged dimensionless units in a type system [33] to support generalization so that, for example, a number of neutrons and a number of protons could be added, resulting in a number of nucleons. Unit one remained as the maximally general (and thus completely uninformative) ‘top type’.

In 2021, Brown reviewed the situation with counted quantities and unit one, expanding on the issues of traceability and realisation of units [34].

Finally, in 2023, Flater described counts as a particular case of *quantal* quantities, which are constrained to exist in integral multiples of a quantum, and argued that the unit used to express a count need not be the same as the quantum (the single entity or event being counted) [35]. The principle of invariance of a quantity to a change of unit then applied to counted quantities and counting units the same as it does to other physical quantities and units.

## 4. Assessment

### 4.1. Dimensions not included in SI

Unit one is indeed recognized as a unit in the 9th edition of the SI brochure [2]. But unlike the base and derived SI units that are listed normally, unit one is a ghostlike entity that manifests only on occasion. It is *not included* in the definition of the SI (section 2.2). It is said to have arisen spontaneously as an entailment of dimensional algebra rather than through a formal decision to adopt it as other units were adopted (section 2.3.3). The reader is *prohibited* from writing it explicitly in a quantitative expression (section 5.4.7). Nevertheless, it plays a unique role in establishing formal traceability to the SI for counted quantities (section 2.3.3)—albeit a vacuous traceability that carries no burden of calibration. For what is presented as an indispensable unit, one receives a confusing treatment.

The importance of treating numerical physical quantities as more than just numbers was fully explained by Reichardt (see the block quote in section 3). When the structure realized by the SI system and quantity calculus is different than the inherent structure of the material world, we have to conclude that the SI structure is either incomplete or wrong. Yet when dimensional analysis suggests that counted quantities, angles, and ratios of two quantities of the same kind are all dimensionless, some evidently conclude that *it must be so* and bravely toss them into the set of real numbers. Section 2.3.3: ‘Such quantities are simply numbers.’ Section 5.4.7: ‘Quantities related to counting… are just numbers.’

The resulting system erases the nature of numerical physical quantities and facilitates two kinds of errors. First, one can make the errors that in every other dimension are recognized as errors of unit conversion; e.g. one can produce an erroneous factor of 2*π* from the confusion of radians with cycles or a factor of eight from the confusion of bits with bytes. Second, one can add, subtract, or convert among quantities of different kinds; e.g. one can add an angle to an amount of data. The risk of confusing different kinds of quantities that happen to be expressed in the same units is not limited to dimensionless quantities, but it is worse because of the sheer number of different kinds that all must refer to the unit one. A simple quantity calculator that implements the system as given will realize de Boer’s assertion ‘All the so-called ‘dimensionless quantities’ belong to one class of quantities of the same kind’ literally and freely convert any dimensionless quantity to any other.

Given the structural difference between a model with a fixed set of dimensions and a world that gives rise to additional dimensions as science progresses, what must yield is the assumption that the set of dimensions enumerated in SI is complete. Quantities that SI does not distinguish are not necessarily the same. The enumeration in the SI brochure of certain dimensions ought not be construed to create difficulties for the use of others in the various domains of science.

### 4.2. Counting units

The problem is exacerbated by rules that effectively ban the appearance of counting units (section 5.4.7). The SI brochure does not allow one to say ‘The throughput is 1 Mb/s’; one is required to shift the unit of data into the description of the quantity and say something equivalent to ‘The bit-throughput is 10^6^/s’. As Mohr and Phillips observed, ‘one cannot do calculations or conversions with phrases that precede the quantity in question’ [22, section 3]; for those working with data, compliance comes at that cost. In practice, computer scientists use bit and byte as units to avoid confusion and enable the expected calculations; compliance with section 5.4.7 would confuse their colleagues and increase the risk of bit–byte conversion errors.

With counting having been used as a measurement method for mass, length, and volume since prehistoric times (e.g. grains of wheat or barley), it is no coincidence that amounts and counts have the same form of expression: a number followed by a unit. When one is specified as the unit associated with a count, it displaces a more informative counting unit that in common usage would be supplied (e.g. 10 passengers, 23 kg/passenger). To a wide audience, ‘23 kg’ and ‘23 kg/passenger’ express different quantities. Suppressing the counting unit invites mistakes to be made.

## 5. Outline of changes to the SI brochure

Proposed is (1) to recognize in the SI brochure that numerical physical quantities are not simply numbers, and (2) to remove from the SI brochure those prescriptions that conflict with common practices in the treatment of dimensionless quantities, especially the definition and use of dimensionless units that are distinguished by kind. The corresponding operational model for quantity calculus has already been described [32, 35].

In the 9th edition of the SI brochure [2], the relevant proposed changes are:

- Section 2.3.3: remove the assertion that any quantity that is defined as the ratio of two quantities of the same kind is simply a number, reduce the emphasis on unit one, and address traceability for dimensionless ratios and counts in a more candid way. For dimensionless ratios, we know that the numerator and denominator will have meaningful traceability if they are considered separately. For counts, we know that some definition for the identification of the entities or events counted must exist, ideally in a standard that is ‘downstream’ [34] of the SI brochure.
- Section 4: acknowledge the role of ‘downstream’ standards for other units and remove the implication that any units not listed in the brochure are ‘unacceptable’ (cf. [36, section 7.5], ‘quantities must be defined so that they can be expressed solely in acceptable units’). The normative goal is to proscribe the use of non-SI units for the dimensions where SI units have been defined; it cannot be to prohibit the definition of units for dimensions that SI does not contain, for which unit one is a meaningless placeholder.
- Section 5.4.2: adjust rules about unit symbols and ‘extra information’ so that they cannot be construed to prohibit the use of dimensionless units beyond unit one (cf. [36, section 7.5], ‘Unacceptability of mixing information with units’).
- Section 5.4.7: remove the assertion that counted quantities are just numbers and allow the use of SI prefixes with counting units other than unit one.
- Section 5.4.8: revise the treatment of angles, in particular the presentation of the equation 1 rad = 1, to catch up with the characterization of the ‘radian convention’ that has appeared in the literature [37].

Those changes would reduce the difficulty of making do without an SI dimension. Whether angles, data, or any other kind of quantity should be allocated an SI dimension should be regarded as a completely separate question.
